# Selling vs. Supporting Motherhood: How Corporate Sponsors Frame the Parenting Experiences of Elite and Olympic Athletes

**DOI:** 10.1177/21674795221103415

**Published:** 2022-06-05

**Authors:** Talston Scott, Sydney V. M. Smith, Francine E. Darroch, Audrey R. Giles

**Affiliations:** 1 Department of Health Sciences, 120862 Carleton University, Ottawa, ON, Canada; 2 School of Human Kinetics, 6363 University of Ottawa, Ottawa, ON, Canada

**Keywords:** pregnancy, parenthood, elite athletes, feminist framing analysis, corporate sponsors

## Abstract

Recently, motherhood and pregnancy in elite sport have received increased attention in sport media. Through a comprehensive news media search across Factiva as well as a gray literature search using Google search engine, we analyzed 115 articles using feminist framing analysis. We developed two primary frames: 1) empowerment versus exploitation, and 2) proactivity versus reactivity. Our results show that many pregnant and parenting athletes frame their respective sponsors as exploitative for recognizing and capitalizing upon their unique marketing value, while these same corporate sponsors frame themselves as industry leaders who empower pregnant and parenting athletes. These two frames show that pregnant/parenting elite athletes commonly face discriminatory policies and practices and that there is often a lack of congruence between marketing and actual corporate practices and policies. These findings arguably reflect larger societal issues related to gender equity and highlight the importance of action over rhetoric to ensure motherhood is supported—rather than marketed—for elite athletes.

Researchers have demonstrated that women’s participation in sport drastically declines after they give birth, and that motherhood has historically signaled the end of professional sporting careers for women (Bialeschki & Michener, 1994; Miller & Brown, 2005; Palmer & Leberman, 2009; Thompson, 1999). Darroch et al. (2019) highlighted the lack of support elite female runners experience during pregnancy and postpartum, with athletes reporting feeling financially penalized by their corporate sponsors for choosing to start families. This finding can be juxtaposed with the marketing and public relations strategies of large athletic apparel companies that sponsor elite athletes and position themselves as advocates for gender equity and supporting women at all stages of their sporting careers (Montaño, 2019). As a greater number of women return to high-level sport after giving birth (International Olympic Committee, 2016), it is a critical time to further our understanding of the impact that elite athletes, corporations, and the media can have on sport, specifically with considerations of maternity as well as pregnancy and parenting policies (Cooky et al., 2013; Darroch et al., 2019; Darroch & Hillsburg, 2017; Fink, 2015).

Drawing on feminist framing analysis, we examine the tensions between the ways in which corporate sponsors frame their support of elite female runners who are pregnant and/or parenting and the ways in which these athletes themselves frame sponsors’ support. This paper is divided into five main sections. First, in the literature review, we contextualize our research by outlining women’s representation in sports and sports media before examining the marketing of elite athlete-mothers and the sponsorship structures within elite athletics. In the second section, we outline our use of feminist framing analysis as well as our approach to data collection using the online news database, Factiva. In the third section we present our results, which yielded two primary frames: 1) empowerment vs. exploitation, and 2) proactivity versus reactivity. We provide further discussion of our findings in the fourth section, followed by the final section of this paper in which we present our conclusions and provide suggestions for future research and the application or utilization of our main findings.

## Literature Review

### Women’s Representation in Sport and Sport Media

Historically, women’s sports have received far less coverage compared to men’s (Bruce, 2016; Creedon, 2014). And, when they do receive coverage, commentators often emphasize women athletes’ physical appearance over their athletic accomplishments (Bruce, 2016; Creedon, 2014; Weber & Carini, 2013). This is especially significant because sports media influences, establishes, and upholds social norms and perceptions that govern sports and athletes, particularly as they pertain to the characterization of female athletes and their experiences (Carter-Francique & Richardson, 2016; Cooky et al., 2013; McGannon et al., 2015). These representations in media construct social meaning and ideology regarding perceptions of women in high-level sport (Carter-Francique & Richardson, 2016; McGannon et al., 2012), which constrain female athletes’ “ability to define themselves in ways that fundamentally alter men’s ideological and institutional control of sport” (Kane et al., 2013, p. 293).

While the limited and gendered coverage of women’s sport has contributed to the perception that motherhood and participation in elite sport are mutually exclusive, this perception has begun to shift over the last decade (Darroch et al., 2019). Although women have and continue to face marginalization through sport, researchers have suggested that women’s participation in sport continues to rise in the developed world (Cooky et al., 2013; Fink, 2015). For example, at the 2016 Rio de Janeiro Olympic Games, 45% of athletes were female, the highest percentage in the Games history at the time (International Olympic Committee, 2016). This record was broken at the 2020 Tokyo Olympic Games, delayed by 1 year due to the COVID-19 pandemic, with nearly 49% of participating athletes being female, representing the first gender-balanced Games in history (Pegoraro & Arndt, 2021). Further, elite athlete-mothers have garnered considerable visibility both in sport as well as off the playing field. For example, McGannon et al. (2015) observed the increased media attention during the 2012 London Olympic Games paid to athlete-mothers.

### Motherhood in Marketing

Corporate sponsors and corporate marketers have begun to respond to these shifting demographic and social trends, albeit in ways that tend to reinforce hegemonic gender roles (Darroch et al., 2019). One viral example of this was Proctor and Gamble’s “Proud sponsor of moms” advertising campaign during the 2012 Olympic Games, which focused on the mothers of Olympic athletes rather than athlete-mothers themselves. The extremely successful campaign highlighted motherhood as being a potentially impactful and significant marketing and public relations theme for advertisers and sponsors (Darroch et al., 2019; Meenaghan et al., 2013).

In March 2021, Nike (2021) released a marketing campaign featuring its new line of apparel, “Nike(M),” a maternity athleisure-wear line designed for pregnant and postpartum individuals. The corporation’s viral advertisement video, titled “The toughest athletes,” featured various professional athlete-mothers including tennis star Serena Williams, United States Women’s National Soccer Team player Alex Morgan, and elite track stars Shelly-Ann Fraser-Pryce, Perri Shakes-Drayton, Nia Ali, and Bianca Williams. With the tagline “Motherhood looks different for everyone. But no matter what you do or how you do it, you are the toughest athlete,” this corporate marketing campaign by Nike further reflected the impact on and significance of the theme of motherhood and pregnancy in elite sport and sport media. Similarly, Adidas, another giant in the athletic apparel industry, has capitalized upon the theme of motherhood in sports marketing, having released a diversity campaign featuring pregnant and breastfeeding mothers (Sowden, 2020).

### Sponsorship Structure and Maternity Protections

Despite increasing presence and representation in sport and media, female athletes continue to face barriers and stressors that their male counterparts do not (Cooky et al., 2013; Fink, 2015). Corporate sponsorship of athletes is not equal, with females receiving fewer and lower paid endorsements than their male counterparts (Coker, 2012; Lobpries et al., 2018; Mogaji, 2019). The organization Women in Sport (2019) reported that a meager 0.4% of total sport sponsorships were allocated to women between 2011 and 2013. Professional runners are unlike other elite athletes in that they do not receive team-based sponsorships and instead sign individual and exclusive contracts with a single sponsor, usually large athletic and shoe companies. This sponsorship structure decreases elite runners’ negotiating and economic capacity and increases their vulnerability to discriminatory language or policies within contracts (Carrillat et al., 2013; Lane, 2012). Furthermore, sponsored athletes are classified as independent contractors rather than company employees, excluding them from benefits such as maternity leave, health coverage, and collective bargaining (Darroch et al., 2019).

In addition to these challenges, female athletes’ contracts often lack explicit provisions and protections for pregnancy, childbirth, or postpartum. This issue is further emphasized by the use of ambiguous language within contracts that can allow a sponsor to terminate or enact financial reductions for nearly any reason, of which pregnancy is often no exception (Darroch et al., 2019). This is concerning for female runners in particular due to the various moving parts of age of peak performance and optimal fertility/childbearing years (Bø et al., 2018) and sponsorship structures. Issues related to contractual obligations have only recently been brought to light likely due to the risks associated with violating non-disclosure clauses in athlete contracts (Darroch et al., 2019).

In May of 2019, the conversation regarding maternity and pregnancy support for elite athletes hit national headlines when *The New York Times* ran an article titled “Nike told me to dream crazy, until I wanted a baby,” in which six-time National USA Outdoor Champion and 2011 World Championship bronze medalist Alysia Montaño (2019) detailed her experiences of dealing with her corporate sponsor, Nike, during pregnancy. Breaking her non-disclosure agreement, Montaño described her unsuccessful fight for maternity protections and for the ability to continue to earn a salary during pregnancy and postpartum, accusing the company of discriminatory practices and policies. Montaño criticized the company for failing to provide maternity supports, and these criticisms were echoed 2 weeks later in a second article in *The New York Times* that featured Allyson Felix (2019), the world’s most decorated track athlete, who revealed a similar experience. Felix, a 13-time World Champion and 11-time Olympic medalist who was one of Nike’s most widely marketed athletes, asked the company to contractually guarantee that she would not be punished for not performing at her best in the months surrounding childbirth. Failing to secure these protections, Felix revealed the lack of congruence between Nike’s marketing and charity initiatives focused on empowering women and the actual practices and policies it implemented.

The public discussions that have been elicited by Felix and other athletes have drawn wide attention toward the (in)congruency between the framing of pregnant/parenting athletes by corporate sponsors and the athletes’ actual maternity and motherhood experiences with their sponsors. Given the very limited research that has examined this discrepancy, we analyzed a variety of media articles using feminist framing analysis to understand the tensions between how sponsoring companies use pregnant/parenting athletes to frame their own social positioning and, in contrast, the ways in which these athletes frame their experiences with their sponsors.

## Feminist Framing Analysis

Feminist framing analysis has been widely used by researchers in the realm of sport communication and media (Bell & Sanderson, 2016), especially by those who have explored media portrayals of female athletes (Bruce, 2016; Jakubowska, 2017; Rightler-McDaniels, 2014; Romney & Johnson, 2020; Seay, 2011; Villalon & Weiller-Abels, 2018; Xu & Kreshel, 2021) and motherhood (Shaw & Giles, 2009). Our use of this approach enabled us to examine the relationships between corporate sponsors and pregnant and parenting athletes while deliberately taking into account the various (and often problematic) ways in which these relationships are framed in the media, including through advertising and corporate marketing.

Goffman (1974) first suggested the idea of frames within a sociological setting as a necessary tactic for organizing information that would otherwise be fragmentary (Rightler-McDaniels, 2014); frames are, in other words, the “schemata of interpretation” (Goffman, 1974, p. 21). But the degree to which one’s organization and interpretation of information can be authentic and thorough is often bounded by the borders of a given frame. Entman (1993) emphasized how frames are defined not only by the various communicable materials that may be included within them but also by those that are omitted. Hence, individuals who create frames (e.g., corporate advertisers, marketers, or journalists) exercise power with those who may be interpreting or perceiving said frames (e.g., a member of society)—an element of power that is stitched into the selective process of inclusion versus omission of information within frames (Entman, 1993).

Adopting the feminist articulation of framing analysis enables researchers to better understand the role of gendered power within the framing process (Hardin & Whiteside, 2010). Feminist scholarship includes, but is certainly not limited to, the examination of gender oppression, gender dynamics, (gendered) power analysis, (gendered) advocacy, and the transformation of society (Carragee & Roefs, 2004; McHugh, 2014). Feminist framing analysis is therefore an ideal framework for understanding how concepts related to what Romney and Johnson (2020) referred to as the “complex duality of feminism and sport” (p. 742) are produced by the media.

Interestingly, if individuals who are responsible for orchestrating company advertisements, marketing, and media productions exercise power in their ability to frame sport in a way that reproduces gender differentiation and gender ideals, then it should also hold true that they are equally in a position to *challenge* gender stereotypes and patterns (Jakubowska, 2017; Kian et al., 2011). For example, Bruce (2016) contended that traditional and dominant discourses of female athletes in online and social media have been newly challenged by emerging discourses that do not construct athleticism and femininity as being incompatible. Simply put, Bruce described female athletes as having been more recently perceived as “pretty *and* powerful” as opposed to merely “pretty *or* powerful” (p. 361). Nevertheless, patterns of gender differentiation in sport repeatedly surface, which is unmistakeably illustrated by the plethora of instances in which men’s sport is consistently presented over that of women’s across a variety of media platforms (Ada Lameiras & Rodriguez-Castro, 2020; Fink, 2015; Peeters & Elling, 2015; Shifflett et al., 2016).

It is important to note that one’s general understanding and interpretation of any given issue or topic—including the damaging perception that sport is a traditionally masculine space and that women are framed as the “other” (Bruce, 2016; Romney & Johnson, 2020)—is shaped and defined by one’s exposure to specific frames (Chong & Druckman, 2007; Kuypers, 2002). In the same vein and what is especially important to acknowledge in this regard is that once a culturally significant topic is defined, it becomes difficult to *redefine* that same issue (Bronstein, 2005; Kian & Hardin, 2009). Hence, tangible insight into the differences in the ways in which corporate sponsors frame messages around pregnancy/parenthood, gender roles, and gender equality compared to how sponsored athletes frame these same topics is integral to the broader understanding of how these issues may be interpreted and defined by society at large.

Feminist framing analysis was thus an appropriate choice for our research as we explored the relationships between corporate sponsors and pregnant/parenting athletes and how these relationships are framed through the media and in advertising. This approach enabled us to specifically consider the ways in which corporate sponsors may exercise power not only with individuals among society who consume their (meticulously framed) advertising and marketing campaigns, but also how they exercise power with the athletes themselves who may be featured within or omitted from these frames. Information can be framed to convey a specific ideology or belief (Zenone et al., 2021)—and framing analysis ultimately permitted us to explore whether the framing processes used by corporate sponsors versus those of sponsored parenting athletes convey similar or opposing beliefs.

## Methods

We conducted a comprehensive news media search across Factiva as well as our gray literature search using the Google search engine. Factiva is a leading online international news database that combines over 30,000 sources from 200 countries (ProQuest, 2021). We selected it as an appropriate database to utilize as the broad range of content provides regional and global insights on business issues and current events. Our additional gray literature search through Google allowed us to further ensure that all relevant sources were included in our search. We included articles in our feminist framing analysis if they were published in English; were dated between November 12, 2017 and May 1, 2021; included discussion of maternity policies, contracts, or experiences in the context of elite running and/or track and field; and included discussion of corporate sponsorship, corporate contracts, or corporate marketing and advertising. The timeline parameter identified for our inclusion criteria was selected because it includes 18 months of coverage prior to the publishing of Alysia Montaño’s *The New York Times* op-ed in May 2019 and extends to include coverage generated as a result of the aforementioned Nike(M) maternity athleisure-wear marketing campaign in March 2021. We excluded articles if they contained no mention of corporate sponsorship or if the primary focus of the article was not on the sport of athletics.

Accessing the Factiva news database, we used the search string “((elite OR professional) adj2 (run* OR athlet*) AND (maternity OR pregnan*) AND (sponsor*)),” which returned a total of 325 results. We downloaded these results into NVivo®, a software program that is commonly used in qualitative and mixed-methods research to organize text, audio, video, and image data for analysis. We applied an identical timeframe using the Google search engine, in which we used the search string “athletics running maternity sponsor.” With this, we identified and downloaded a total of 130 articles into NVivo, which represented the first 13 pages of Google search results. We excluded results beyond the first 13 pages due to decreased relevancy and accuracy in relation to our search.

**Figure 1.**
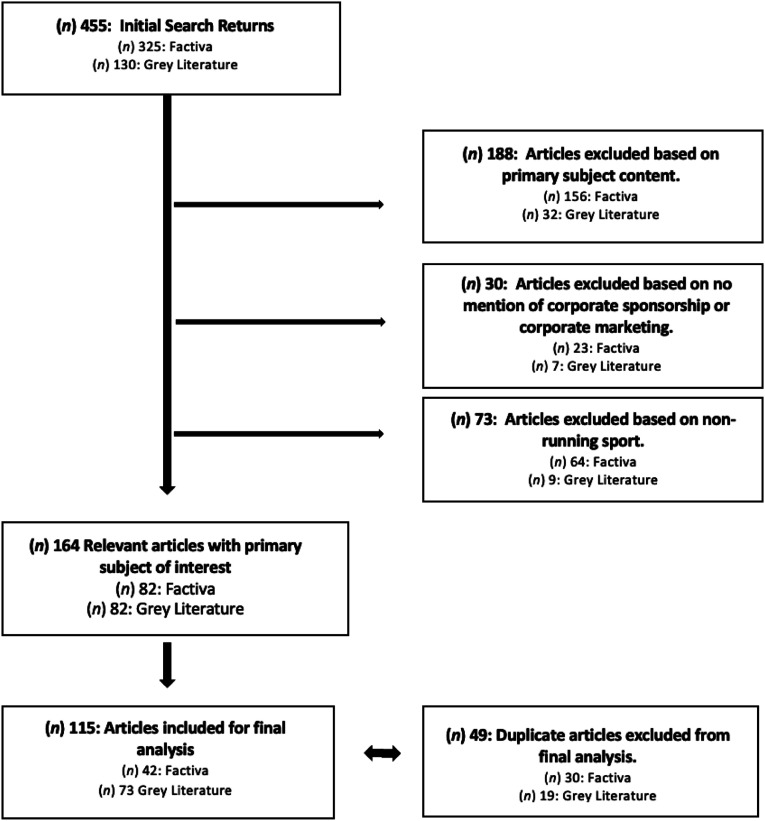
Article Screening Process. *Note.* Overview of article screening process used in determining articles for inclusion for analysis.

Based on non-relevant primary subject content, we excluded 188 articles (*n* = 156 from Factiva, *n* = 32 from gray literature). We excluded a further 73 articles based on non-running sport content (*n* = 64 from Factiva, *n* = 9 from gray literature), and we excluded another 30 articles based on there being no mention of corporate sponsorship nor marketing (*n* = 23 from Factiva, *n* = 7 from gray literature). There were 164 relevant articles that remained, of which a final 115 were included for analysis after screening for duplicates (*n* = 40 Factiva duplicates, *n* = 9 gray literature duplicates). An overview of the article screening process is provided in Figure 1.

## Results

The first author coded the articles in NVivo. The codes were then reviewed and verified by all authors. We generated two main frames from the codes: (1) empowerment versus exploitation, and (2) reactivity versus proactivity. Coded units included in the first frame were “sponsorship contract,” “discrimination,” “negative contractual change,” “pressure to train/return to competition,” “punish/penalize,” and “marketing value as pregnant and parenting athletes.” Coded units we included in the second frame were “calls to action,” “positive contractual change,” “pregnancy/maternity policy,” “positive social change,” and “references to Montaño’s 2019 *The New York Times* op-ed.” These two frames show that pregnant and parenting elite athletes commonly face discriminatory policies and practices. While our analysis primarily refers to the experiences of sponsored athletes at Nike, the lack of support for mothers is a systemic issue within the industry more broadly. The practice of suspending or voiding contracts due to pregnancy was repeatedly described as an industry-standard practice. A majority of the articles included for analysis focused on sponsored athletes within the United States, particularly Alysia Montaño and Allyson Felix. It is noteworthy that the athlete-mothers leading this movement are Black women, especially considering the historic marginalization of Black women’s contributions to social change (Lindsey, 2020; Mirza, 2009).

### Exploitation Versus Empowerment

Our results show that many pregnant and parenting athletes frame their respective sponsors as exploitative for recognizing and capitalizing upon their unique marketing value, while these same corporate sponsors frame themselves as industry leaders who empower pregnant and parenting athletes. Negative contractual changes—including termination, suspension of contract, or reductions in salary for any reason (with no exceptions for childbirth)—were reported as occurring frequently as a result of an athlete becoming pregnant. For example, in a video that accompanied her *New York Times* op-ed, Montaño revealed that “when I told [Nike] I wanted to have a baby during my career, they said, ‘simple, we’ll just pause your contact and stop paying you’” (Pells & Graham, 2019, para. 13). Similarly, when Allyson Felix (2019) was in the process of renegotiating her Nike contract following the high-risk birth of her daughter, she noted that “the corporation wanted to pay me 70% less, despite all my victories” (para. 7). Felix also acknowledged the actions of two of her former Nike teammates, Alysia Montaño and distance runner Kara Goucher: “they told stories we athletes know are true, but have been too scared to tell publicly: If we have children, we risk pay cuts from our sponsors during pregnancy and afterward” (para. 3).

In the articles we reviewed, athletes also reported that vague and ambiguous language is frequently used in contracts in addition to industry-standard, strict, non-disclosure agreements. These contractual elements have resulted in athletes feeling arbitrarily punished for becoming pregnant. Terms such as “punish,” “penalize,” and “punitive” were commonly used by athletes to frame their experiences navigating contractual re-negotiations and their relationships with corporate sponsors when becoming pregnant (e.g., Adler, 2019; Felix, 2019; Kai, 2019). For example, Montaño expressed this sentiment,

The word pregnancy [in a contract] feels like it’s a target on a woman. It was devaluing in the mind of the sports industry to become a mother. We shouldn’t have to be fearful of that. We are not devalued because we become pregnant. We are not damaged goods. (MacKenzie, 2019, para. 6)

Furthermore, sponsored athletes explicitly described feeling not only punished but also discriminated against for choosing to start families. This discrimination was illustrated by Montaño questioning “why, just because I am a woman and an athlete should I be penalized for acting on a basic human right? Men don’t lose out on money when they become fathers” (Got You Girl, 2019, para. 4). This discrimination is not limited to elite athletics only, as Montaño also noted that “in industries across the board, we are faced with the motherhood penalty” (Dokoupil, 2021, p. 3).

Throughout the articles we reviewed, pregnant and parenting athletes also reported feeling pressured to train and compete during pregnancy and to return quickly to competition following childbirth to meet contractual obligations. This pressure to return to competition is directly related to the financial penalty that is a result of the structure of corporate sponsorship contracts for professional runners, which notably lack pregnancy and maternity considerations. Elite long-distance runner and World Championship medalist Kara Goucher described this pressure:

I felt like I had no choice. Looking back, I wish I had said, “Fuck you.” But I was the sole income provider for our family at that time and I felt so much pressure to get back to competing. You are being held hostage in the situation you’re in. (Adler, 2019, para. 8)

Goucher also detailed the physical and psychological impacts this pressure to return to competition had on her:

I felt like I had to leave him [her newborn infant son] in the hospital, just to get out there and run, instead of being with him like a normal mom would. Returning to competition so quickly was a bad choice for me. And looking back and knowing that I wasn’t the kind of mother that I want to be – it’s gut-wrenching. (Montaño, 2019, para. 9)

This type of pressure has also led pregnant athletes and others to express concern over increased injury risk and the potential long-term health impacts on athletes and their children. Los Angeles-based writer Chelsea Steiner (2019) highlighted that the structure of corporate sponsorship contracts “force[s] female athletes to remain competitive well into their pregnancy, and to resume training almost immediately after giving birth to ensure their livelihoods – at the risk of their own health and the health of their children” (para. 5). This risk was similarly highlighted and evident when Goucher detailed how “today, I have chronic hip issues because I kept getting stress fractures as a result of rushing back when my body wasn’t healed” (Adler, 2019, para. 17).

These experiences result in physical, emotional, and financial distress for sponsored athletes who become pregnant and stand in stark contrast to the marketing and media campaigns that are used by corporate sponsors. Sponsors frame themselves as industry leaders in women’s empowerment and equity through their use of campaigns that reflect a clear recognition of the unique marketing value that pregnant and parenting athletes contribute. Several athletes described the marketing value of pregnant and parenting athletes as being exploited by corporate sponsors to socially position themselves and their associated brand as champions for women and for mothers. In her *New York Times* op-ed, Montaño (2019) described a “multi-billion-dollar industry that praises women for having families in public – but doesn’t guarantee them a salary during pregnancy or early maternity” (para. 8). Similarly, Kara Goucher summarized the chasm between Nike’s advertising and the reality she faced when choosing to start a family:

Nike’s advertising is amazing. I get chills when I watch their videos, but I have to say to myself, remember when you got pregnant, and they just stopped paying you? That’s the same company! Nike promoted me during my pregnancy – it was selling this image of motherhood – while causing me so much stress. (Adler, 2019, para. 20)

Lindsay Crouse, co-author of the aforementioned *New York Times* Montaño op-ed, underscored the unique value that pregnant and parenting athletes contribute: “the company knows these athletes’ value goes beyond racing” (Crouse, 2019, para. 3). This was especially evident in the corporate marketing and experience of Kara Goucher who “didn’t learn until after she was already pregnant with her son in 2010 that Nike wouldn’t pay her until she began racing again despite making twelve unpaid appearances for the brand during her high-risk pregnancy” (Haney, 2019, para. 10). Goucher’s negative experience in this regard was strikingly highlighted by the fact that she “had to wait four months to even announce her pregnancy so that Nike could (conveniently for them) publish the news on Mother’s Day” (DiversityInc, 2019, para. 7).

This apparent hypocrisy is further amplified by the social positioning and values that many athletic apparel companies, especially Nike, claim to champion through their advertising. In her *New York Times* op-ed, Montaño (2019) wrote,

Many athletic apparel companies, including Nike, claim to elevate female athletes. A commercial released in February received widespread acclaim for spotlighting women at all stages of their careers, from childhood to motherhood. On Mother’s Day this year, Nike released a video promoting gender equality. But that’s just advertising. (para. 1)

These examples collectively raise crucial questions and concerns around corporate sponsors’ handling of elite female runners’ public expressions of their experiences of exploitation, specifically with regard to how this has led the companies to situate themselves within the changing culture of parenthood within elite athletics.

### Reactivity Versus Proactivity

Corporate sponsors utilize marketing and media to frame themselves as proactive companies that support female athletes throughout their athletic careers, including pregnancy and motherhood. In contrast, pregnant and parenting sponsored athletes frame the corporate and cultural change within sporting and sport sponsorship as the result of, and reaction to, prominent athletes sharing their experiences of discrimination. Despite changes made to policy and practices within the industry (e.g., Nike specifically now contractually guarantees an athlete’s pay plus bonuses for 18 months surrounding pregnancy (Chavez, 2019)), there have been no public acknowledgments of wrongdoing or apologies/compensation to athletes whose careers and earning potential were negatively impacted.

This represents a problematic situation of reactivity against proactivity as sponsored athletes and commentators alike have highlighted that the positive changes seen within the industry are the result of companies *reacting* to prominent athletes speaking out and pushing back—not the result of companies taking action in *proactive* ways. Sport journalist Amanda McCracken (2020) noted that the two *New York Times* op-eds by Montaño and Felix “led to a public outcry and a congressional hearing, and now athletic apparel companies, including Nike, are beginning to adjust their maternity policies to protect female athletes” (para. 4). Reacting to the Nike(M) campaign, Goucher wrote,

I appreciate that they have made changes, but I have received no apology or acknowledgement from Nike on how I and many other mother athletes were treated – or received the pay they withheld from me. But I am happy to see the improvement and hopefully the next generation won’t have to suffer the way I, and so many other athletes, did. (Women’s Running, 2021, para. 7)

Similarly, responding to the updated policies that Nike announced for pregnant and parenting athletes, Montaño noted,

Yes. We want Nike to sponsor athletes and support them through pregnancy, and thereafter, but we want them to acknowledge the fight and the struggle that it took to get them to make a change. We DO NOT WANT them to use our women to make money and while doing so forcing their athletes that have been mistreated to post advertisements as a way of sweeping their struggles under the rug. (Hambleton, 2021, para. 7)

In response to the newest Nike(M) advertising campaign, Colleen Quigley, an American 2016 Olympic steeplechaser and former Nike-sponsored athlete, expressed her support for athletes speaking out:

Can’t watch this [Nike(M)] ad without thinking of the women who had to suffer under Nike in order to become mothers before [Felix] decided to put her foot down and demand change. *It feels important to me that people know who gets the credit for this progress to value moms within Nike* [emphasis added]*.* (Women’s Running, 2021, para. 11)

This sentiment was expressed commonly by pregnant and parenting sponsored athletes in the articles we reviewed, who publicly shared their feelings of frustration concerning their sponsors’ public relations and advertising campaigns. While the campaigns prominently lauded and advertised athlete-mothers, in reality they were provided with little to no actual tangible supports or resources, and changes in maternity and pregnancy policies were only the result of athletes breaking non-disclosure agreements, speaking out, and risking future financial security.

## Discussion

Our study builds on the important work of several other scholars who have explored the role of sponsorship (e.g., Darroch et al., 2019; Meenaghan et al., 2013) as well as media representations of women and mothers in sport in general and elite running in particular (e.g., Bruce, 2016; McGannon et al., 2012, 2015). Our findings emphasize how major athletic apparel brands’ framing of themselves as sponsors as well as their framing of the culture around parenthood in elite running were in stark contrast to the sponsored athletes’ framing and their calls for recognition of the vital roles they have played within the current developing chapter of sport. Our results reveal and raise further questions around the power that corporate sponsors and apparel companies exercise (Cornwell, 2014, 2020), the marginalization of Black women’s contributions to social change (Lindsey, 2020; Mirza, 2009), and gender roles within sport (Bruce, 2016; Fink, 2015).

### Exploitation Versus Empowerment

There is an increasing need to understand the very critical role that media play in sponsorship as media platforms continue to evolve in this day and age (Meenaghan et al., 2013). We found a distinct divergence in how pregnant/parenting athletes framed sponsoring companies as exploitative while these same brands framed themselves as empowering to athlete-mothers. One may argue that it is indeed preferred that a multinational corporation as big as Nike be engaged in such conversations rather than simply turning a blind eye and, for this reason, it is imperative that we acknowledge the significance of the fact that discussions around pregnancy and parenthood within elite running are happening at all. Yet, we would be remiss to not recognize the immense difference between *advertising* and *advocating*. Based on our results, the advertising strategies used by corporate sponsors paint a picture of not only support but also encouragement of the uniting of parenthood and athletics, while the athletes’ framing of these same topics suggest that these acts of support are largely promoted in theory but have yet to materialize in practice.

The approach that corporate sponsors have seemingly adopted—assigning less importance to improving the lives of pregnant/parenting athletes and greater emphasis to their own advancements in the marketing industry—is a course of action that is problematic on many fronts (Darroch et al., 2019; Rasmussen et al., 2021). While this tactic diminishes the significance of the many issues that are at the very core of any pertinent discussions in this regard (particularly those surrounding gender equity in sport), it also severely muffles the voices of the athletes who have spoken up to advocate for change. In particular, the disavowal of the role of two Black women, Felix and Montaño, in creating change furthers a history of Black women’s contributions to social change being ignored (Lindsey, 2020; Mirza, 2009). This damaging approach is evidence of the problematic dynamics that are often present in sport sponsorship, owing in large part to the stringent influence of sponsors’ corporate objectives (Schönberner et al., 2021).

The relationship between a sponsored athlete and a sponsor is typically characterized by an athlete depending on a sponsor for “financial viability” (Cornwell, 2014, p. 15), which is a form of dependence that can lead to a power imbalance and can consequently influence the attitudes and behaviors of those involved in the relationship (Cornwell, 2020). Though the athletes included in this research have taken risks by speaking out against corporate sponsors, many athletes have remained silent. By virtue of contracts and non-disclosure clauses, sponsors are hindering athletes’ ability to express their opinions and are thus further reflecting and reinforcing the existing power imbalance (VeneKlasen et al., 2002) that often exists between sponsored elite athletes and athletic apparel companies. Through the disregard of athlete contributions to the positive cultural shift, sponsors are—once again—exerting their power.

### Proactivity Versus Reactivity

The culture around pregnancy, parenthood, elite running, and sponsorship is beginning to shift—but at the expense of genuine accountability and acknowledgment toward the ground-breaking steps that leading athletes have taken. In speaking out and sharing their experiences, pregnant/parenting athletes have brought this topic and pertinent conversations to mainstream media and have pushed the boundaries of what is considered standard in sponsorship contracts. These athletes’ actions have also led to the reputations of several apparel companies having been publicly questioned and thus potentially damaged, causing companies to engage in reactions that are consistent with Benoit’s (1997) image repair crisis communication. In hopes of restoring their reputations in the face of challenges against their handling of athletes’ experiences around pregnancy and maternity, major corporate sponsors such as Nike have begun to introduce and publicly advertise relevant incremental policy changes. Though these progressive measures are indeed a pivotal part of the cultural shift, we found that such acts are tainted by the absence of any apology, recognition, or acknowledgment of the experiences that athletes faced. Companies have framed themselves as the helping hands who offer considerable levels of support to female athletes through pregnancy and motherhood and, in turn, have portrayed a message to the broader public that depicts the noticeably positive cultural shift within the sports industry as being a result of their own initiatives.

Sport occupies a unique position within advertising as a result of the strong emotional connections that consumers express toward athletes, teams, or a sport in general (Pyun & James, 2011), enabling athletic apparel companies to severely influence the messaging around how gender is perceived in sport (Rasmussen et al., 2021). Our findings highlight how the compelling advertising by companies coupled with refusal to credit athletes who took great personal risk in speaking out against maternal discrimination within athletics has significant impacts on how society broadly views and understands gender roles within sport, while also further perpetuating the problematic framing that corporate sponsors attempt to utilize and capitalize upon. This response and continued trend call our attention to the detrimental effects this sort of disingenuous brand activism can have in dictating representations within the media and the sports industry more broadly, all the while appropriating social justice movements without revealing and/or addressing misaligned practices and policies (de Oca et al., 2020; Rasmussen et al., 2021).

The results of our framing analysis allowed us to recognize the significant discrepancy that exists between how elite runners who are pregnant/parenting present their relationships with corporate sponsors versus how these relations are framed by the companies themselves. Though this has allowed us to address an important gap in the literature on this topic, there are still limitations to this study. Our use of Factiva and Google may have limited our ability to access a more broad and diverse set of articles. As a result, our results and analysis were focused solely on how high-profile athletes like Allyson Felix and huge corporate sponsors such as Nike have framed conversations around maternity support and athlete-sponsor relationships. Our results are thus lacking an understanding in terms of the ways in which other sponsored and professional runners may experience or perceive discussions around pregnancy/parenthood in relation to elite running, as well as additional brands that may be lost in the shadows behind more dominant names like Nike.

Furthermore, our framing analysis enabled us to explore various materials that sponsored athletes and athletic apparel companies choose to include and share publicly, whether it be through advertising, online op-eds, or social media; however, our analysis only captured information that is in the public domain. While this may be considered a limitation of our study, it also draws our attention toward further questions around the nature of any information that has been omitted (whether it be purposefully or not) from all relevant frames.

There are numerous avenues of future research. Given the nature of sponsorship, contracts, and confidentiality agreements between athletes and corporate sponsors, it is likely that there are many conversations around pregnancy/parenthood that occur behind closed doors. There is a need for a thorough investigation into both elite athlete-parents’ as well as corporate sponsors’ perspectives with regard to the changing culture of parenthood in relation to elite running, athletes’ experiences (beyond those that are publicly available) around navigating parenthood within the elite running world, and how these insights may come into play within athlete-sponsor relationships. Furthermore, there is a dearth of academic literature and, in turn, a severe lack of knowledge around elite athlete-fathers in particular and their roles within, perspectives on, and insights into the culture of parenthood within elite running. Though there are important differences between women’s and men’s roles in childbirth and parenthood, this does not mean that men are simply not affected. Yet, advertising and marketing campaigns around elite athletics and parenthood are almost exclusively geared toward mothers. It is thus imperative that a greater and more diverse accumulation of athlete voices be sought and heard, as well, including those of fathers and the LGBTQIA2+ community.

## Conclusion

Overall, our research has highlighted the divides in the ways pregnant and parenting sponsored athletes and corporate sponsors frame the relationships governing their interactions and sponsorship contracts. While some sponsored athletes have called attention to the exploitative nature of these relationships and the reactive responses seen within the industry following public disclosure and backlash, corporate sponsors frame themselves as empowering women at all stages of their career and as proactive industry leaders who advocate for gender equity within sport. Despite these tensions, the athletic industry is beginning to see changes in the culture around parenthood and elite running, as evidenced by contractual policies and protections being implemented for pregnant and parenting athletes. This positive cultural shift is further evidenced by the work Alysia Montaño has continued to do since turning the spotlight to the barriers and bias that professional athlete-mothers face through her *New York Times* op-ed. Montaño went on to found *&Mother*, a non-profit organization that works to affect real world change in the ongoing effort of supporting a woman’s choice and ability to pursue both a career and motherhood. In November of 2021, &Mother (2021), in collaboration with Oiselle, a leading athletic apparel brand focused on reimagining what is possible for female athletes within corporate sponsorship and the sports industry more broadly, released a model framework for “Sponsorship Contract Provisions for Pregnancy and Parental Leave.” By designing and publishing this framework, the women who executed this initiative have provided a new standard for contract language to protect and support sponsored athletes who are either pregnant or parenting.

While a significant portion of our analysis and discussion is focused on Nike specifically, it is paramount to note that these are industry-wide problems that arguably reflect larger societal issues relating to gender equity, with the sporting industry acting as just one example where these social problems manifest. In the pursuit of gender equity within the sporting industry and society more broadly, it is critical to recognize, address, and mitigate the barriers that pregnant and parenting athletes face and—most importantly—to strive to create a culture where motherhood is actually supported, not just sold through advertising.
